# Impact of Environmental Indicators on the COVID-19 Pandemic in Delhi, India

**DOI:** 10.3390/pathogens10081003

**Published:** 2021-08-09

**Authors:** Sherry Mangla, Ashok Kumar Pathak, Mohd. Arshad, Doyel Ghosh, Prafulla Kumar Sahoo, Vinod Kumar Garg, Ubydul Haque

**Affiliations:** 1 International Institute for Population Sciences, Mumbai 400088, Maharashtra, India; sherry@iipsindia.ac.in; 2 Department of Mathematics and Statistics, Central University of Punjab, Bathinda 151401, Punjab, India; doyel11111996@gmail.com; 3 Department of Mathematics, Indian Institute of Technology Indore, Simrol, Indore 453552, Madhya Pradesh, India; arshad@iiti.ac.in; 4 Department of Statistics and Operations Research, Aligarh Muslim University, Aligarh 202002, Uttar Pradesh, India; 5 Department of Environmental Science and Technology, Central University of Punjab, Bathinda 151401, Punjab, India; pk.sahoo@cup.edu.in (P.K.S.); vinodkgarg@cup.edu.in (V.K.G.); 6 Department of Biostatistics and Epidemiology, University of North Texas Health Science Center, Fort Worth, TX 76177, USA; mdubydul.haque@unthsc.edu

**Keywords:** air pollutants, COVID-19, Delhi, humidity, rainfall, temperature, wind speed

## Abstract

Currently, there is a massive debate on whether meteorological and air quality parameters play a crucial role in the transmission of COVID-19 across the globe. With this background, this study aims to evaluate the impact of air pollutants (PM_2.5_, PM_10_, CO, NO, NO_2_, and O_3_) and meteorological parameters (temperature, humidity, wind speed, and rainfall) on the spread and mortality due to the COVID-19 outbreak in Delhi from 14 Mar 2020 to 3 May 2021. The Spearman’s rank correlation method employed on secondary data shows a significant correlation between the COVID-19 incidences and the PM_2.5_, PM_10_, CO, NO, NO_2_, and O_3_ concentrations. Amongst the four meteorological parameters, temperature is strongly correlated with COVID-19 infections and deaths during the three phases, i.e., pre-lockdown (14 March 2020 to 24 March 2020) (r = 0.79), lockdown (25 March 2020 to 31 May 2020) (r = 0.87), and unlock (1 June 2020 to 3 May 2021) (r = −0.75), explaining the variability of about 20–30% in the lockdown period and 18–19% in the unlock period. NO_2_ explained the maximum variability of 10% and 7% in the total confirmed cases and deaths among the air pollutants, respectively. A generalized linear model could explain 80% and 71% of the variability in confirmed cases and deaths during the lockdown and 82% and 81% variability in the unlock phase, respectively. These findings suggest that these factors may contribute to the transmission of the COVID-19 and its associated deaths. The study results would enhance the ongoing research related to the influence of environmental factors. They would be helpful for policymakers in managing the outbreak of COVID-19 in Delhi, India.

## 1. Introduction

The outbreak of the novel coronavirus (COVID-19), associated with Severe Acute Respiratory Syndrome Coronavirus 2 (SARS-CoV-2), began in December 2019 in Wuhan, China. The disease has been affirmed to have human-to-human transmissibility [1], which elevated colossal attention in China and worldwide [2]. Due to its devastating effects worldwide, COVID-19 was declared a global pandemic by the World Health Organization [3]. In India, the first case of COVID-19 was reported on 30 January 2020, in Kerala. The instances in India started increasing at a higher pace, more exponentially with each passing day. Until the beginning of May 2021, there were more than 20.66 million confirmed cases of COVID-19 and around 226,000 deaths in India, reported on the WHO official portal for COVID-19 [4]. Delhi, the largest commercial city of North India, is one of the worst affected cities due to COVID-19 in India, with over 1.21 million cumulative infected cases and more than 17,000 casualties as of 3 May 2021 [5]. The first case of the COVID-19 pandemic in the national capital was reported on 2 March 2020, when an older person from East Delhi with a travel history to Italy tested positive for COVID-19 [6].

Delhi is the ninth most populated metro city globally, with a population of 20 million. Located in Northern India, the national capital territory covers an area of 1484 km^2^, making it the largest city in terms of area in the country. Delhi possesses a dry winter and humid subtropical climate, bordering on a hot semi-arid climate. The average annual rainfall is approximately 886 mm, mostly falling out during the monsoon months of July through August. The maximum and minimum temperature in the city ranges between 2 °C and 47 °C, except for −2.2 °C and 48.4 °C, which are the lowest and highest temperatures, respectively [7]. It is one of the most polluted cities in the country, with the most increased volumes of airborne particulate PM_2.5_, which is considered the most hazardous particulate to health, with 153 micrograms [8]. The rising levels of air pollution have significantly increased lung-related diseases, especially asthma and lung cancer, among children and women in Delhi [9,10]. COVID-19 is also a respiratory disease directly transmitted by close contact through respiratory droplets emitted from an infected person [11].

Recently, several studies from various parts of the world reported that meteorological/weather conditions have a crucial role in the spread of COVID-19 [1,12,13,14]. A study by Zoran et al. (2021) provides evidence that climate parameters, such as temperature, humidity, and wind speed, can trigger the transmission of COVID-19 in Spain [15]. Tosepu et al. (2020) reported that the average temperature is positively correlated with the COVID-19 pandemic in Jakarta, Indonesia [16]. Similarly, an investigation in Singapore revealed that temperature and dew point positively impact daily and cumulative COVID-19 cases [17]. Bolaño-Ortiz et al. (2020) reported enhanced airborne transmission by wind speed due to a correlational existence [18]. In another study, Şahin (2020) analyzed the impact of population and weather parameters on COVID-19 in Turkey [19], and reported a strong correlation among them. An association between the transmission of COVID-19 and environmental factors was also demonstrated by Muhammad et al. (2020) in New York, USA [20]. In India, studies from the states of Maharashtra and Punjab discussed the role of environmental factors in the spread of COVID-19 during different phases of the pandemic, reporting significant correlations between environmental variables and COVID-19 cases [21,22].

There is still an insufficiency of data in several COVID-19 hotspots in Delhi. Since the research related to environmental indicators in COVID-19 is still contradictory, this study will provide rigorous insight to understand this relationship effectively. More vigorous studies must understand these factors to improve forecasting models that can be effective for public health measures and examine the COVID-19 pandemic in Delhi, India, and elsewhere. Thus, the study was conducted in Delhi to analyze the impact of air pollutants (PM_2.5_, PM_10_, CO, NO, NO_2_, and O_3_) and meteorological parameters (temperature, humidity, wind speed, and rainfall) on new infections and mortality due to the COVID-19 outbreak.

## 2. Materials and Methods

### 2.1. Data Collection

**Figure 1:**
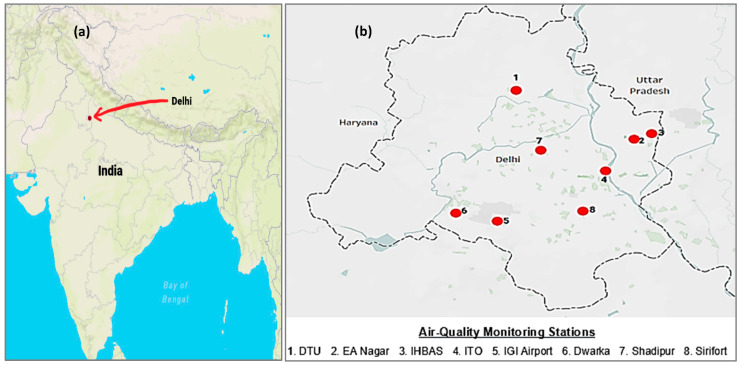
(**a**) Map showing the location of the study area of Delhi, India. (**b**) Map showing the outline of the study area and the location of eight air quality and weather monitoring stations in Delhi, India.

The analysis was carried out in the national capital of India, Delhi, as displayed in Figure 1a. The data for environmental indicators were extracted from the Ministry of Environment, Forest, and Climate Change for the Government of India. It comprised concentrations of PM_2.5_ (µg/m^3^), PM_10_ (µg/m^3^), NO (µg/m^3^), NO_2_ (µg/m^3^), CO (mg/m^3^), ozone (µg/m^3^), temperature (°C), humidity (%), wind speed (m/s), and rainfall (mm) from 14 March 2020 to 3 May 2021. These parameters’ data were collected for eight real-time air quality monitoring stations in Delhi, specifically under the Central Pollution Control Board (CPCB) [23]. The substations were selected so that they geographically became an effective representative of the national capital, covering North, South, East, West, and Central Delhi (Figure 1b). While analyzing these parameters, the average for the eight substations was considered for effective representation. The time series data on COVID-19 cumulative infections and deaths in Delhi were taken for the same period, i.e., 14 March 2020 to 3 May 2021, using a reliable, crowdsourced database repository [5]. The study period starting from 14 March 2020 was considered based on data availability and the need to incorporate the different phases of COVID-19 lockdown. Furthermore, the data of all the environmental and climate indicators, as well as COVID-19 occurrences, were classified into different phases of COVID-19 based on restrictive nationwide policies, such as the pre-lockdown phase (14–24 March 2020), lockdown phase (25 March–31 May 2020), and the unlock phase (1 June 2020–3 May 2021).

### 2.2. Spearman’s Correlation Test

Due to the lack of normality in the dataset, we employed Spearman’s rank correlation for studying the relationship between air pollutants, climate factors, and the impact of COVID-19 in Delhi during 14 March 2020 through 3 May 2021. A correlation matrix was calculated to describe the relationship between all the parameters and other components. The mathematical formula for Spearman’s correlation coefficient is given by:

$$r_{S}=1-6\;\frac{\sum_{i=1}^{n}d_{i}}{n\left(n^{2}-1\right)}\;$$

where n is the number of observations and d_i_ is the difference of the rank between two variables.

### 2.3. Generalized Linear Model

A generalized linear model was employed to analyze the extent of variability by the various air pollutants and the meteorological parameters in COVID-19 cases and deaths. In real life, the assumptions of normality and constant variance are not satisfied by the dataset, hence, a simple linear regression model is challenging to apply. The GLM is a unification of both linear and non-linear regression models that incorporates non-normal response distributions. Estimates with a *p*-value < 0.05 were observed to be significant during the analysis. R^2^ values were used as a measure of variability that a model explains. Furthermore, adjusted R^2^ values were calculated for the complete models using the formula:

$$Adjusted\;R^{2}=1-\left(1-R^{2}\right)*\frac{n-1}{n-k-1}$$

where n = sample size and k = number of independent variables.

The location maps in Figure 1a, b have been prepared using Tableau version 2020.4.5. The statistical analysis in the paper was performed using Microsoft Excel and R version 4.0.2.

## 3. Results

**Figure 2:**
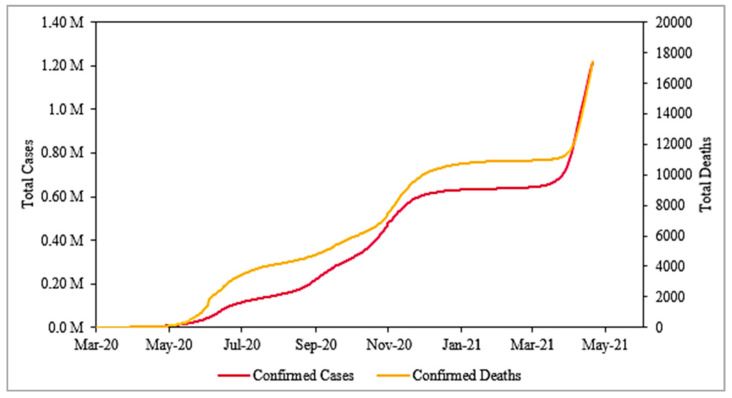
Impact of COVID-19 in Delhi, India from 14 March 2020 to 3 May 2021.

Daily COVID-19 infections and deceased cases are presented in Figure 2. This displays a speedy growth of COVID-19 in Delhi, starting from seven confirmed cases on 14 March 2020 until 24 March i.e., the beginning of lockdown, and then rapidly rising to 19,844 cumulative cases by the end of lockdown. Similarly, the death toll also increased rapidly. By the first week of May 2021, there were as high as 1.21 million cumulative infections and a total of 17,414 deaths.

**Figure 3:**
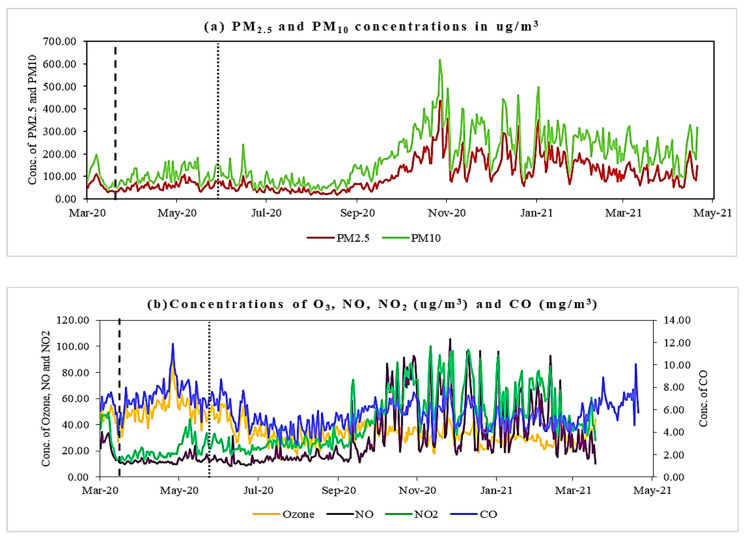
(**a**) PM_2.5_ and PM_10_ concentration (µg/m^3^); (**b**) concentrations of O_3_, NO, NO_2_ (ug/m^3^), and CO (mg/m^3^) from 14 March 2020 to 3 May 2021 in Delhi. The two dotted lines separate three lockdown periods: pre-lockdown, lockdown, and unlock, consecutively.

The concentrations of PM_2.5_, PM_10_, NO, NO_2_, CO, and O_3_, taken from 14 March 2020 to 3 May 2021, are presented in Figure 3a,b. Additionally, the dependence between the air pollutants and COVID-19 incidence and mortality is examined using the Spearman rank correlation. Matrices for the three phases, i.e., pre-lockdown, lockdown, and unlock, are displayed in Table 1, Table 2 and Table 3. The correlation coefficients of very few pairs of parameters turned out to be significant before the lockdown (Table 1). The coefficient in the lockdown phase between the cumulative cases and PM_2.5_, (r = 0.60), PM_10_ (r = 0.62), NO_2_ (r = 0.65), and CO (r = 0.53) share a high positive correlation with a *p*-value < 0.05. These parameters are also positively correlated with the cumulative deaths during the lockdown period (Table 2). The findings suggested a significant correlation between COVID-19 cases (and deaths) and the parameters PM_2.5_ (r = 0.57), PM_10_ (r = 0.57), NO (r = 0.48), NO_2_ (r = −0.25), CO (r = 0.11), and O_3_ (r = −0.19) in the unlock phase (Table 3).

**Figure 4:**
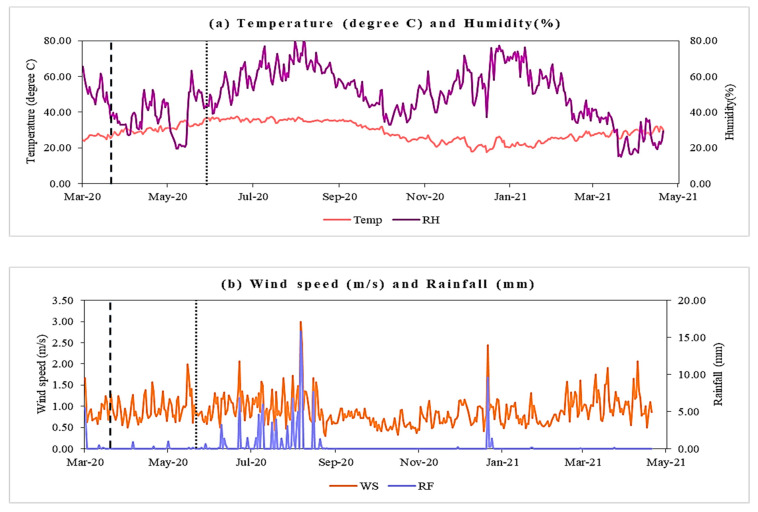
(**a**) Temperature (°C) and humidity (RH) and (**b**) wind speed (WS) (m/s) and rainfall (RF) (mm) from 14 March 2020 to 3 May 2021 in Delhi. The two dotted lines separate three lockdown periods: pre-lockdown, lockdown, and unlock, consecutively.

**Figure 5:**
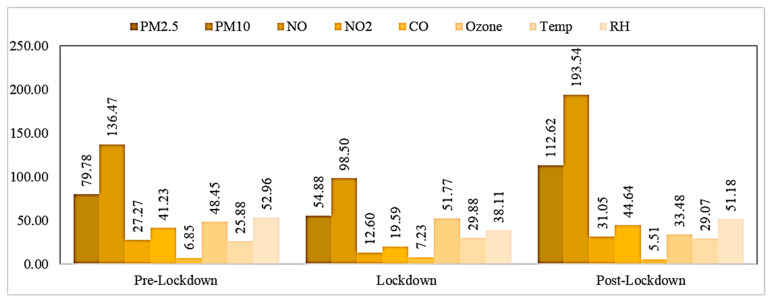
Average concentrations’ change of different parameters (during pre-lockdown, lockdown, and post-lockdown period) in Delhi.

The variation in meteorological parameters during the pre-lockdown, lockdown, and unlock phases is shown in Figure 4a,b. In Delhi, summer starts in early April and peaks in late May or early June. Average temperatures near 38 °C are followed by monsoons that last until mid-September. Winter begins in November and peaks in January with an average temperature of around 6–7 °C and ends by the first week of March. Humidity is at its maximum during the monsoons in Delhi, and otherwise remains low to moderate [24]. Spearman correlation results in Table 1 show that only temperature and humidity were the significantly correlated variables with confirmed cases (r = 0.79; −0.85) in the pre-lockdown period. Temperature was strongly correlated during the lockdown (r = 0.87) and unlock period (r = −0.65) with the COVID-19 infections and deaths (Table 2 and Table 3). Temperature was also significantly correlated with the air pollutants, such as PM_2.5_, PM_10_, NO, and NO_2_, during the lockdown and unlock periods. The factors of wind speed and rainfall did not seem to have a good correlation with COVID-19 in this study. The correlation between relative humidity and COVID-19 cases and deaths was −0.85 in the pre-lockdown period, −0.31 in the lockdown period, and −0.43 in the unlock period, all significant for a *p*-value < 0.05. Figure 5 displays that the average concentrations of all the air pollutants showed the following trends: PM_2.5_ in lockdown < PM_2.5_ in pre-lockdown < PM_2.5_ in unlock; PM_10_ in lockdown < PM_10_ in pre-lockdown < PM_10_ in unlock; NO in lockdown < NO in pre-lockdown < NO in unlock; NO_2_ in lockdown < NO_2_ in pre-lockdown < NO_2_ in unlock; CO in unlock < CO in pre-lockdown < CO in lockdown; O_3_ in unlock < O_3_ in pre-lockdown < O_3_ in lockdown.

Furthermore, to understand the variability by different predictors, the generalized linear model was constructed for the lockdown and unlock periods. The results for these models are presented in Table 4 and Table 5, respectively. The GLM model for pre-lockdown was omitted from the analysis because of an insufficient data quality and higher insignificance in correlations. For modeling, total confirmed cases and total deaths were dependent variables, with the other air pollutants and the climate parameters as independent variables. NO_2_, among other pollutants, explained maximum variability in total cases (10%) and deaths (7%) for the lockdown period. Other air pollutants did not seem to contribute much to the transmission of the virus. For instance, PM_2.5_ and PM_10_ explained only 1% of the variability in the confirmed cases. At the same time, PM_10_ failed to explain any variation in total deaths during the lockdown. NO explained only 2% variation in the infections and deaths in the lockdown, and around 2% variability in the unlock period. Unlike the lockdown model, O_3_ explained a total variability of 16% in total cases and deaths, followed by CO (13%; 12%), PM_2.5_ (2%), and PM_10_ (1%) during the unlock phase. The complete models for confirmed cases in the lockdown showed an adjusted R^2^ value of 80%, whereas, for the deaths, it was 71%.

In contrast to this, the models in the unlock phase yielded an adjusted R^2^ value of 82% and 81%, respectively, for total cases and deaths. Out of all the parameters, including air pollutants and meteorological factors, temperature significantly explained maximum variability for cumulative cases and deaths in both the lockdown (23%; 31%) and unlock periods (18%; 19%). Humidity, rainfall, and wind speed played no role in explaining the variability in COVID-19 transmission.

## 4. Discussion

### 4.1. Association of Air Pollutants with COVID-19 Cases and Deaths

Figure 3a,b show that most air pollutant levels were drastically reduced during the lockdown phase until the end of August. Although the air pollutants PM_2.5_, PM_10_, NO_2_, and CO have shown a strong positive correlation with COVID-19 incidences, this may not be true on the ground, as, during the lockdown, all the pollutants were drastically reduced. The most significant reduction is seen in the concentrations of PM_2.5_, PM_10_, NO_2_, NO, and CO. A similar observation was also reported from other megacities in India [25,26,27] and elsewhere [28,29].

PM_2.5_, PM_10_, NO_2_, and O_3_ concentrations increased drastically after the lockdown. Exposure to such air pollutants is harmful to the respiratory and cardiovascular systems in humans [30]. COVID-19 infection is related to the respiratory system. Vulnerability to such pollutants would increase the risk of deaths due to COVID-19. According to Yamada et al. (2020), an increase of 1% in long-term exposure to PM_2.5_ results in a 5.7% increase in COVID-19 mortality [31]. Similar results suggested by Dales et al. (2020) show a significant association between increased PM_2.5_ and NO_2_ levels and daily COVID-19 deaths [32]. However, in an ideal situation, the escalation in pollutant levels would have correlated COVID-19 infection and mortality and air pollutants more robustly than in the lockdown period. However, this was not the case on the ground. Even if the pollutant emission increased, it took a little while for the situation to normalize in terms of people’s movement for work or other affairs, so the correlation levels with COVID-19 dipped instead of rose. Moreover, during the lockdown, the COVID-19 infections and deaths were positively correlated with PM_2.5_ and PM_10_ (*p*-value < 0.05). However, this exposure is not necessarily related to COVID-19 conditions [31]. The increase in COVID-19 cases in Delhi might result from more underlying factors. For instance, a mass migration of people from city centers to hometown and rural areas was caused by excessive job loss and fear of lockdowns. Furthermore, the effect of unfavorable meteorological conditions needs more research [33,34].

The average concentration plot of different air pollutants (Figure 5) shows that O_3_ and CO concentrations also increased in the lockdown period in Delhi. Zhao et al. (2020) reported an increase of 47% in O_3_ concentrations during the lockdown period in mainland China [35]. Similar reporting in O_3_ concentrations was observed in many European cities [36]. Ozone production is dependent on various factors. The anthropogenic emissions and volatile organic compounds (VOCs) are the primary precursors for O_3_ generation. In addition to these pollutants, the meteorological parameters also play an essential role in the production of O_3_. Advection of warm and polluted air masses can also raise the near-surface O_3_ concentrations [37,38,39].

### 4.2. Relationship with the Meteorological Variables

Climate parameters, such as temperature, humidity, and wind speed, are reported to be vital factors in the transmission of SARS-CoV2 [15]. Ma et al. (2020) reported a significantly positive relation between daily temperature and deaths due to COVID-19, and a negative correlation for relative humidity [14]. A similar result is obtained in this study. COVID-19 cases and deaths in the lockdown period positively correlate with temperature and negatively correlate with humidity throughout the study period. In low humidity, the moisture in the exhaled bioaerosols evaporates rapidly. It forms droplet nuclei that may remain in the air for a more extended period, facilitating the increased pathogen transmission [40].

Low humidity can reduce the airway cilia cells’ ability to remove virus particles, thereby exposing the host to the virus [41]. These associations indicate that the human body is at a higher risk of infection by SARS-CoV-2 in high temperature and low humidity environments. However, various studies suggest that the weather variables (especially temperature) seem to have a more negligible effect on the transmission of COVID-19. A study reports no correlation between temperature and humidity with COVID-19 incidences [42]. At the same time, others suggest that there is no supporting evidence that the COVID-19 transmission will decline in warm temperatures [43].

### 4.3. Determining Factors of COVID-19 Cases and Deaths

The GLM model findings explain that the role of particulate matter (PM_2.5_ and PM_10_) or other pollutants, such as NO, CO, and O_3_, in the transmission of the virus is quite negligible in the lockdown period. CO and ozone seem to have contributed quite well to the model with confirmed cases and deaths in the unlock period by explaining maximum variability among all the other air pollutants. The pessimistic estimate values for O_3_ in Table 5 suggest that with an increase in O_3_ concentration, the number of confirmed cases and deaths decreases. It may be due to the virucidal act of the ozone on the host defense. It has previously been reported that O_3_ is hugely influential in disinfection and sterilization against many respiratory infections, like influenza and SARS-CoV-1 viruses [44]. The adjusted R^2^ values for all four models vary in a noticeable pattern. The values for models with confirmed cases in the lockdown and unlock period give a better adjusted R^2^ value. It can be inferred from this that the GLM models provide a better estimate for the confirmed cases than for the deaths. The estimates of temperature in the model for confirmed cases in lockdown hold a positive relationship. After the lockdown, it is negative. The lockdown period comprises March, April, and May, typically known as the summer season in India. As per the estimates of the GLM model (Table 4), COVID-19 incidences seem to increase with increasing temperature considering this period. The temperature in India after May begins to vary (Figure 4a) with the onset of monsoon season (June–September) slightly, followed by autumn (October–November), and finally winter (December–February) to summers starting in March. Therefore, the estimates from this period in the GLM model (Table 5) suggest that the total infection and deaths from COVID-19 decrease in the said temperature variation. The parameters of wind speed and rainfall have the most minuscule contribution in explaining any variability or being correlated with total cases or deaths.

### 4.4. Limitations

This study does not include certain factors, such as individual human behavior, recent mass gatherings, or new COVID-19 variants, that might influence the spread of COVID-19 and its associated mortality. There is also the unavailability of data for these measures at the regional level, especially in India. Given the data, more complex research can be done to incorporate these measures and understand the extent of COVID-19 in Delhi. Another limitation is that the study used aggregated data rather than for the individual. The prime focus was to study the impact of environmental indicators under different restriction phases imposed to control COVID-19 transmission. Therefore, the findings are ecological.

## 5. Conclusions

The present study favors the argument that the COVID-19 lockdown has significantly helped clean the air environment. A reduction in the levels of PM_2.5_, PM_10_, NO, and NO_2_ was observed during the lockdown. This is because of the stringent conditions for the movement of vehicles and other kinds of restrictions. However, the concentration of O_3_ increased during the lockdown, which is possible because it was enforced during the warmer months in Delhi i.e., April and May. In warm temperatures, ozone pollution is expected to increase. Besides this, the positive correlation between PM_2.5_, PM_10_, and CO concentrations with COVID-19 incidences needs more research to understand its mechanism. This research finds that increasing temperature and decreasing humidity may increase daily new infections and deaths due to the coronavirus. At the same time, other meteorological and air pollutants exhibit no significant relation with the COVID-19 pandemic. The GLM models suggest that the temperature is statistically a substantial contributor to the spread of the virus, but this could also be related to seasonal variations in the Indian capital. It is also found that the air pollutants and meteorological parameters in this study could correlate better with the confirmed infections than deaths in Delhi. This could be because the deaths due to a disease would depend more on the health infrastructure and affordable medical facilities, primarily not on these factors. Considering the current situation of the COVID-19 pandemic in Delhi, policy measures, such as imposing lockdown restrictions and reducing contact rates, are suggested to be helpful to control the spread. Therefore, the impact of these factors may be considered in policy development to control the COVID-19 pandemic.

## Figures and Tables

**Table 1:** Spearman’s correlation coefficient during pre-lockdown i.e., between 14–24 March 2020.

Pre-Lockdown	PM_2.5_	PM_10_	NO	NO_2_	CO	Ozone	Temp	RH	WS	RF	Confirmed Cases
PM_2.5_	1										
PM_10_	0.95 *	1									
NO	0.64 *	0.75 *	1								
NO_2_	0.55	0.69 *	0.87 *	1							
CO	0.52	0.41	0.42	0.26	1						
Ozone	0.15	0.06	0.17	0.02	0.91 *	1					
Temp	0.51	0.38	–0.08	–0.22	0.33	0.21	1				
RH	−0.15	−0.05	0.39	0.44	−0.39	−0.45	−0.53	1			
WS	−0.16	−0.21	−0.28	−0.06	−0.64 *	−0.73 *	−0.28	0.25	1		
RF	−0.35	−0.35	−0.19	−0.29	−0.49	−0.39	0.10	0.54	0.10	1	
Confirmed Cases	0.08	−0.07	−0.47	−0.60	0.33	0.43	0.79 *	−0.85 *	−0.33	−0.08	1

* p < 0.05.

**Table 2:** Spearman’s correlation coefficient during lockdown i.e., between 25 March–31 May 2020.

Lockdown	PM_2.5_	PM_10_	NO	NO_2_	CO	Ozone	Temp	RH	WS	RF	Confirmed Cases	Confirmed Deaths
PM_2.5_	1											
PM_10_	0.92 *	1										
NO	0.58 *	0.48 *	1									
NO_2_	0.83 *	0.74 *	0.77 *	1								
CO	0.40 *	0.43 *	−0.17	0.24 *	1							
Ozone	0.27 *	0.31 *	−0.27 *	0.12	0.98 *	1						
Temp	0.64 *	0.68 *	0.40 *	0.67 *	0.42 *	0.34 *	1					
RH	−0.61 *	−0.61 *	−0.43 *	−0.62 *	−0.28 *	−0.19	−0.37 *	1				
WS	−0.39 *	−0.31 *	−0.34 *	−0.36 *	0.08	0.16	0.05	0.31 *	1			
RF	−0.35 *	−0.32 *	−0.05	−0.25 *	−0.17	−0.09	−0.08	0.46 *	0.35 *	1		
Confirmed Cases	0.60 *	0.62 *	0.31 *	0.65 *	0.53 *	0.46 *	0.87 *	−0.31 *	0.20	−0.02	1	
Confirmed Deaths	0.60 *	0.62 *	0.31 *	0.65 *	0.53 *	0.46 *	0.87 *	−0.31 *	0.20	−0.02	0.99 *	1

* *p* < 0.05.

**Table 3:** Spearman’s correlation coefficient after lockdown i.e., between 1 June 2020 to 3 May 2021.

Unlock	PM_2.5_	PM_10_	NO	NO_2_	CO	Ozone	Temp	RH	WS	RF	Confirmed Cases	Confirmed Deaths
PM_2.5_	1											
PM_10_	0.98 *	1										
NO	0.81 *	0.81 *	1									
NO_2_	0.86 *	0.85 *	0.94 *	1								
CO	0.38 *	0.40 *	0.18 *	0.25 *	1							
Ozone	−0.04	−0.03	−0.22 *	−0.16 *	0.84 *	1						
Temp	−0.76 *	−0.71 *	−0.73 *	−0.80 *	−0.10	0.22 *	1					
RH	−0.27 *	−0.35 *	−0.10	−0.18 *	−0.53 *	−0.42 *	0.09	1				
WS	−0.46 *	−0.47 *	−0.54 *	−0.52 *	−0.26 *	−0.04	0.20 *	0.00	1			
RF	−0.54 *	0.56 *	−0.39 *	−0.45 *	−0.39 *	−0.21 *	0.41 *	0.54 *	0.31 *	1		
Confirmed Cases	0.57 *	0.57 *	0.48 *	−0.25 *	0.11 *	−0.19 *	−0.65 *	−0.43 *	0.03	−0.44 *	1	
Confirmed Deaths	0.57 *	0.57 *	0.48 *	0.54 *	0.11 *	−0.19 *	−0.65 *	−0.43 *	0.03	−0.44 *	0.99 *	1

* *p* < 0.05.

**Table 4:** Variability in COVID-19 confirmed cases and deaths during the lockdown.

Parameter	Confirmed Cases (Lockdown)			Confirmed Deaths (Lockdown)		
	R^2^ Adjusted (Complete Model) = 0.80			R^2^ Adjusted (Complete Model) = 0.71		
	β	*p*-Value	R^2^	β	*p*-Value	R^2^
PM_2.5_	−90.48	0.07	0.01	−2.10	0.09	0.02
PM_10_	23.14	0.25	0.01	0.12	0.80	0.00
NO	−781.93	0.01 *	0.02	−14.95	0.04 *	0.02
NO_2_	806.80	0.00 *	0.10	13.83	0.00 *	0.07
CO	−6124.62	0.01 *	0.02	−87.26	0.16	0.01
O_3_	761.21	0.02 *	0.02	8.93	0.26	0.01
TEMP	1634.56	0.00 *	0.23	39.01	0.00 *	0.31
RH	149.85	0.00 *	0.04	2.50	0.02 *	0.03

* *p* < 0.05.

**Table 5:** Variability in COVID-19 confirmed cases and deaths after the lockdown.

Parameter	Confirmed Cases (Unlock)			Confirmed Deaths (Unlock)		
	R^2^ Adjusted (Complete Model) = 0.82			R^2^ Adjusted (Complete Model) = 0.81		
	β	*p*-Value	R^2^	β	*p*-Value	R^2^
PM_2.5_	−2834.1	0.00 *	0.02	−49.00	0.00 *	0.02
PM_10_	1519.2	0.00 *	0.01	27.40	0.00 *	0.01
NO	−5138.3	0.00 *	0.02	−75.45	0.00 *	0.02
NO_2_	−2613.3	0.01 *	0.00	−29.76	0.03 *	0.00
CO	347546.1	0.00 *	0.13	4695.55	0.00 *	0.12
O_3_	−50344.1	0.00 *	0.16	−705.31	0.00 *	0.16
TEMP	−35350.1	0.00 *	0.18	−496.73	0.00 *	0.19
RH	−1577	0.00 *	0.00	−2.76	0.79	0.00

* *p* < 0.05.

## Data Availability

Data are available upon request. Please contact author for data requests.
